# Real-Life Assessment of Multi-Pollutant Exposure and Its Impact on the Ocular Surface: The Bike-Eye Pilot Study on Urban Cyclists in Bologna

**DOI:** 10.3390/ijerph22121818

**Published:** 2025-12-04

**Authors:** Roberto Battistini, Natalie Di Geronimo, Emanuele Porru, Valeria Vignali, Andrea Simone, Suzanne Clougher, Silvia Odorici, Francesco Saverio Violante, Luigi Fontana, Piera Versura

**Affiliations:** 1 DICAM, Alma Mater Studiorum Università di Bologna, 40126 Bologna, Italy; roberto.battistini2@unibo.it (R.B.); valeria.vignali@unibo.it (V.V.); andrea.simone@unibo.it (A.S.); 2 Ophthalmology Unit, IRCCS Azienda Ospedaliero-Universitaria di Bologna, 40138 Bologna, Italy; natalie.digeronimo@aosp.bo.it (N.D.G.); luigi.fontana6@unibo.it (L.F.); 3 Occupational Medicine Unit, DIMEC, Alma Mater Studiorum Università di Bologna, 40138 Bologna, Italy; emanuele.porru2@unibo.it (E.P.); francesco.violante@unibo.it (F.S.V.); 4 Ophthalmology Unit, DIMEC, Alma Mater Studiorum Università di Bologna, 40138 Bologna, Italy; suzanne.clougher@gmail.com (S.C.); silvia.odorici2@studio.unibo.it (S.O.)

**Keywords:** ocular surface, particulate matter, tear fluid biomarkers, air pollution exposure, electron microscopy, scanning, environmental exposure, urban health

## Abstract

**Background**: Urban air pollution, particularly fine particulate matter (PM2.5 and PM10), poses health risks, including damage to the ocular surface. This pilot study (BIKE-EYE) aimed to assess ocular exposure to airborne pollutants during bicycle commuting and to evaluate particle presence in human tear fluid. **Methods**: Fifteen healthy volunteers wore portable sensors measuring PM2.5 and PM10 during daily bike commutes over six months. Exposure was calculated as time-weighted integrals over the ten days preceding an ophthalmologic exam assessing conjunctival hyperemia, epithelial damage, tear film quality, and meibomian gland function. Ocular symptoms were assessed via the Ocular Surface Disease Index (OSDI). Tear samples were analyzed using scanning electron microscopy (SEM) and energy-dispersive X-ray spectroscopy (EDS). **Results**: Higher pollutant exposure was significantly associated with conjunctival hyperemia and corneal epithelial damage, while temperature and humidity showed no effect. OSDI scores moderately correlated with PM levels. SEM/EDS analysis confirmed airborne particles in post-exposure tear samples, including carbonaceous material, aluminosilicates, iron, and sulfur compounds. **Conclusions**: Ocular surface alterations and conjunctival hyperemia were significantly associated with air pollution exposure, while subjective symptoms showed weaker trends. The detection of particulate matter in human tear fluid supports the use of the ocular surface as a sensitive, non-invasive tool for biomonitoring. These findings highlight its potential role in early warning systems for pollution-related health effects, with implications for public health surveillance and urban planning.

## 1. Introduction

Urban areas have evolved into complex, multifunctional spaces shaped by transportation networks, walkable surfaces, and cycling infrastructure that facilitate daily mobility and social interaction. In this context, sustainable urban planning and active mobility—such as walking and cycling—are increasingly recognized for their positive impact on population health and environmental quality [1,2]. Features like street connectivity, safe cycling lanes, and accessible urban green spaces have been shown to promote physical activity and reduce exposure to air pollution [2,3]. These aspects are central to current public health and climate mitigation strategies across Europe and globally [4,5]. As cities transition toward more sustainable and “smart” models, integrating health-oriented mobility policies remains a crucial challenge for the design of livable urban environments [1,5].

Bologna’s bicycle mobility infrastructure is particularly suitable for flat regions like Emilia-Romagna. Recent data based on 320,000+ cycling trips (Bella Mossa, April–September 2017) confirm regular usage patterns and seasonal variation, demonstrating the city’s consolidated bike culture [6]. As part of the Metropolitan Sustainable Urban Mobility Plan (PUMS), Bologna is implementing the “Biciplan” and expanding its Bicipolitana network with ongoing and planned infrastructure—over 420 km built or under construction, with an additional ≈149 km financed—to improve connectivity and daily bike usage [7]. Monitoring strategies include GPS tracking via cycling challenges (e.g., Bella Mossa) and flow detection through bicycle counters, enabling data-driven assessment of bikeway use and mobility behavior [6,8].

While active mobility helps reduce future pollutant emissions, existing urban air pollution still poses significant health risks during physical activity in cities. These effects can be assessed using the WHO Health Economic Assessment Tool (HEAT), which quantifies mortality risks and health benefits resulting from walking and cycling in relation to air pollution exposure [9,10]. Key air pollutants affecting quality of life (QoL) include particulate matter (PM), volatile organic compounds (VOCs), nitrogen and sulfur oxides (NO_x_, SO_x_), carbon oxides (CO, CO_2_), and ozone (O_3_). Chronic exposure to these substances has been linked to respiratory and cardiovascular disease and is estimated to cause approximately two million premature deaths annually worldwide [11].

Emilia-Romagna is recognized as one of Europe’s hotspots for air pollution. In 2010 alone, PM10 and PM2.5 were estimated to cause 4.4 and 2.8 deaths per 100,000 inhabitants, respectively, in the region [11]. Beyond respiratory and cardiovascular effects, air pollutants can impact the ocular surface, leading to redness, irritation, tearing, foreign body sensation, and blurred vision. Unlike the respiratory mucosa, the eye allows for direct, non-invasive examination, positioning it as a potentially sensitive indicator of environmental health risks [12,13,14]. However, the pathophysiological mechanisms by which airborne pollutants interact with the tear film, cornea, and conjunctiva remain incompletely understood. Loss of immune tolerance and activation of pro-inflammatory cytokines appear to be involved, but further investigation is needed.

In addition to inorganic pollutants, aerosols include biological particles such as pollen and fungal spores, collectively referred to as primary biological aerosols (PBAs) [15]. These components can trigger allergic rhino conjunctivitis and asthma in sensitized individuals, impairing quality of life and reducing productivity [16]. PBAs may also release allergenic proteins (aeroallergens) into the atmosphere, which can interact with inorganic pollutants and enhance their clinical impact [17,18]. Climate change is expected to further influence the duration, intensity, and timing of aeroallergen exposure, compounding the health burden in urban environments [19]. For these reasons, airborne pollutants and allergens are monitored through institutional networks using high-precision samplers. However, such equipment is typically expensive and stationary, limiting its ability to reflect individual exposure during daily activities [20,21].

The monitoring of inorganic air pollutants has recently been transformed by the emergence of low-cost, portable smart sensors capable of measuring pollutant concentrations in real time and integrating the data into accessible databases [21]. This technological shift enables the development of automated, user-level air quality monitoring systems that provide data both suitable for scientific analysis and accessible for public health awareness. Several scientific studies have applied such sensors in real-life commuting scenarios, including bicycle-based air quality monitoring employed in dynamic exposure assessment and citizen science projects across Europe [19,20,21]. 

Although several projects over the past decade have estimated individual exposure to organic and inorganic aerosols and correlated them with self-reported inflammatory symptoms, no published study to date has combined personal exposure assessment to inorganic pollutants with direct objective measurements of resulting ocular surface inflammation. In particular, the direct detection and characterization of particulate matter in human tear fluid remains largely unexplored. However, a few preliminary investigations have applied Schirmer strips coupled with scanning electron microscopy (SEM) and energy-dispersive X-ray spectroscopy (EDS)**,** revealing both biological and inorganic particles—including metals, minerals, and silicates—on the ocular surface in tear samples [22,23].

The primary aim of the BIKE-EYE project was to assess the short-term effects of real-life exposure to urban air pollution on the ocular surface, with particular focus on the conjunctiva—the only exposed mucosal tissue of the human body. Given its accessibility and sensitivity, the ocular surface may serve as an early and non-invasive sentinel for environmental damage. This pilot study enrolled a limited number of healthy individuals to evaluate the feasibility of combining wearable air quality monitoring with detailed ophthalmologic assessments and tear sample analysis. The results from this initial cohort are intended to inform the design of larger-scale investigations and support the development of personalized strategies for exposure risk assessment and ocular health monitoring in urban settings.

## 2. Materials and Methods

Fifteen healthy adult volunteers were recruited from the staff of the University of Bologna and were regular users of the Alma Bike bicycle-sharing initiative (University of Bologna project). The study followed strict inclusion criteria: participants did not wear contact lenses, had not undergone ocular surgery within the past year, and had no known allergies or systemic diseases that might affect ocular surface health. Sampling was conducted between May and June 2023. This pilot study enrolled a small convenience sample of 15 participants from a real-life university bike-sharing program, reflecting the demographic profile at the time of recruitment. Although the limited sample size and gender imbalance do not allow for generalization, no formal power analysis was performed, as the study was primarily intended to assess feasibility and provide proof of concept for the combined use of personal exposure sensors and ocular surface evaluation. The collected data serve to explore preliminary trends and inform the design of future, larger-scale studies. All participants provided written informed consent, and the study protocol was approved by the Bioethics Committee of the University of Bologna (protocol number 0173473, 28 July 2022), in accordance with the Declaration of Helsinki.

*Clinical evaluation*—This was performed in a single morning visit after at least six months of bike commuting and pollutant monitoring, with participants arriving at the eye clinic directly by bicycle. The ophthalmologic assessment included slit lamp examination with grading of conjunctival hyperemia using the Efron scale [24]; evaluation of ocular surface damage through vital staining, scored according to the NEI scale, with a range from 0 to 5 for the cornea and 0 to 18 for the conjunctiva [25]; tear quantity measured with the Schirmer test and tear film stability assessed through non-invasive Break-Up Time (NiBUT), following the methodology proposed by the Diagnostic Methodology Subcommittee of the International Dry Eye Workshop [26]; measurement of blink rate and inter-blink interval (IBI), along with calculation of the Ocular Surface Protection Index (OPI), a global indicator defined as the ratio of NiBUT to IBI, where values below 1 reflect incomplete protection between blinks [27]; infrared meibography to assess the condition of the lower eyelid meibomian glands, expressed as a percentage of gland loss [28]; and evaluation of ocular discomfort symptoms through the 12-item Ocular Surface Disease Index (OSDI) questionnaire [29], including its three subscales—ocular symptoms, vision-related functioning, and environmental triggers—as well as a Numerical Rating Scale (NRS) for pain, ranging from 0 (no pain) to 10 (worst pain). Both instruments demonstrated good reliability in detecting symptom changes and were included in the statistical analysis.

*Tear Sampling and SEM-EDX Analysis.* Tear samples from three individuals were analyzed to assess the feasibility of particulate detection and characterization by SEM-EDX. Because the particle counting procedure is not automated and highly time-consuming, the analysis was carried out as a preliminary feasibility assessment on this limited dataset.

Tear collection was performed in a real-life setting to preserve natural tear film composition. Participants arrived at the ophthalmology clinic by bicycle, having commuted directly from home after their usual morning hygiene, including standard facial washing. No additional ocular decontamination was performed prior to tear sampling. The right eye was arbitrarily selected for particulate detection. Tear fluid was collected using a micropipette fitted with a sterile disposable tip. Participants were instructed to tilt their head laterally, bringing the ear toward the corresponding shoulder, and to maintain this position for approximately two minutes to promote tear pooling at the lateral canthus. Twenty microliters of tear fluid per eye was gently aspirated, avoiding contact with the ocular surface, and immediately deposited in the central area of sterile polycarbonate membrane filters (Millipore, pore size 0.1 µm, VCTP01300) for scanning electron microscopy (SEM) and subsequent elemental analysis.

Samples were allowed to air-dry at room temperature for 20 min under a glass bell to minimize airborne contamination. To monitor potential environmental or procedural contamination, blank control filters were prepared by depositing Milli-Q system water microfiltered at 0.25 µm onto polycarbonate membranes using the same micropipette and sterile disposable tips employed for tear collection. These filters were dried under identical conditions and processed in parallel with the tear samples, undergoing the same mounting, coating, and imaging steps, thereby serving as environmental and procedural background controls. In addition to these laboratory controls, a tear sample collected after thoroughly rinsing the ocular surface with sterile saline was included as a physiological negative control and processed identically, allowing assessment of the intrinsic baseline particulate signal and helping to distinguish true environmental deposition from sampling artifacts.

Filters were mounted on aluminum stubs using double-sided conductive tape and coated with a 10 nm chromium layer using a sputter coater. SEM imaging was performed using a Zeiss EVO MA10 microscope (Carl Zeiss S.p.A Italia, Milano, Italy), operated at an accelerating voltage of 15 kV and a working distance of 9 mm to optimize resolution and depth of field. All samples were examined at a fixed magnification of 200×, and for each eye, 20 randomly selected, non-overlapping fields were analysed. The elemental composition of selected particles was determined by Energy Dispersive X-ray Spectroscopy (EDS).

Image processing and particle characterization were performed using ZEISS ZEN CORE EM 3.9 image analysis software, which allowed semi-automatic detection and measurement of individual particles. Pre-processing included the standardization of contrast and gamma and the application of a uniform lookup table (LUT) across all images to ensure comparability. For each identified particle, area, minimum and maximum Feret diameters (Feret min/max), and circularity (4π·Area/Perimeter^2^) were calculated as primary morphometric descriptors. Data were processed and summarized using GraphPad Prism (v. 8) to evaluate particle size distribution and basic descriptive statistics.

*Pollution data harvesting—methodology*—Cyclists were recruited among university staff through a dedicated call, with selection criteria prioritizing participants with a high frequency of weekly home-to-work commuting by bicycle. Each selected participant was provided with a Smart Citizen Kit (SCK) and instructed to carry the sensor during all urban trips. Participation in the ophthalmologic assessment was conditional upon consistent use of the SCK for at least ten consecutive days prior to the clinical visit.

To enable real-time pollutant measurement during urban bicycle commuting, a customized low-cost sensor kit, scattered light model, was developed in collaboration with FabLab Barcelona (https://fablabbcn.org/) building upon the Smart Citizen Kit (SCK) platform. The SCK comprises a modular set of hardware components designed for environmental monitoring, with a focus on scalability, reproducibility, and user accessibility. The architecture centres around a data-logging module (the *Data Board*), which provides network connectivity and supports both the Smart Citizen Kit and Smart Citizen Station configurations. This core module is based on an ARM Cortex-M0+ 32-bit SAMD21 microcontroller, operating at 48 MHz and running the Smart Citizen Firmware. The processor combines low power consumption with adequate performance, featuring 32 KB of RAM and 256 KB of flash memory. The sensor kit used in this study included:

- The Smart Citizen sensor module with integrated GPS and antenna.
- A USB charging cable and power supply.
- A custom 3D-printed protective enclosure for mounting on bicycles.

The system also integrates software components, including a cloud-based data storage platform and a sensor data analysis framework, enabling real-time data transmission, visualization, and analysis.

The specific sensor models employed for pollutant detection (e.g., PM2.5, PM10, temperature, humidity) are detailed in Table 1.

The SCK is integrated by a high-quality GPS NEO-M8U GPS Breakout from Sparkfun 2 (SparkFun Electronics, Niwot, CO, USA). The NEO-M8U module is a 72-channel u-blox M8 engine GNSS receiver, meaning it can receive signals from the GPS, GLONASS, Galileo, and BeiDou constellations with ~2.5-m accuracy. The module supports concurrent reception of three GNSS systems. The combination of GNSS and integrated 3D sensor measurements on the NEO-M8U provide accurate, real-time positioning rates of up to 30 Hz. Compared to other GPS modules, this breakout maximizes position accuracy in dense cities or covered areas. Even under poor signal conditions, continuous positioning is provided in urban environments and is also available during complete signal loss (e.g., short tunnels and parking). Lock time is further reduced with on-board rechargeable battery; there is a backup power enabling the GPS to get a hot lock within seconds.

The pollutants monitored included PM2.5 and PM10, which represent a heterogeneous mixture of solid and liquid airborne particles. These parameters were selected due to their recognized health relevance and because they are subject to specific regulatory thresholds in the Emilia-Romagna region. Under typical urban conditions, PM derives from both natural and anthropogenic sources, with road traffic being a primary contributor—particularly through pavement wear, brake abrasion, and tire degradation.

*Sensors validation and calibration*—Several tests were carried out on the sensor kit: indoor particulate testing; outdoor vs. static dynamic comparison; outdoor dynamic comparison, indoor particulate testing. These tests were conducted prior to the main indoor analyses to assess the difference between each case and free air sampling. In particular, the tests performed in sensors post production phase are: Indoor particulate tests, Outdoor dynamic vs. static comparison, Outdoor dynamic comparison, as illustrated in online documents sources of SparkFun_u-blox_NEO-M8U (SparkFun Electronics Niwot, CO, USA) [30].

On field calibration—A specific calibration has been carried out to compare data collected by sensors with ARPA (Regional environmental Agency) station. The criteria of ARPA station are very different, because the harvesting is through a filter installed on the top of the station, while optic sensors detect pollutants through light scattered criteria. Anyway, a sampler sensor has been placed in close proximity to the station (in the very center of Bologna, in front of main boulevard, at Porta San Felice, Bologna) and two days of detection has been defined in April 2023. The results demonstrate that PM2.5 detected by sensors have an offset of 20% in medium less than the data detected by Station, while PM10 detected by sensors presents a wide offset, about 42% less than the data detected by Station. It is also relevant to underline that sensors are constructed to detect pollutants in dynamic conditions, during the run, and not to be statically places, as in the case of that calibration.

*Exposure estimation*—Exposure levels to airborne pollutants were estimated following established methodologies from the literature. This approach builds on the foundational work of Sexton and Ryan [31], who outlined general strategies for human exposure assessment, and the concept of “pollutant dose” introduced by Ott [32]. The analytical model applied here is also consistent with the recommendations of Monn [33], particularly regarding indoor/outdoor exposure distinctions. Exposure was calculated as the time-weighted integral of PM2.5 and PM10 concentrations recorded during urban cycling activities, as illustrated in F. Specifically, data were retrieved from the ten days preceding the ophthalmologic visit, and exposure was expressed as PM2.5 or PM10 × time (h·µg/m^3^). To improve the accuracy of exposure estimates, a correction factor of 0.69 was applied to account for the potential contribution of indoor air pollution and to isolate outdoor exposure, as suggested by Monn [32]. The calculation method is expressed as:

$$EXP(PM2.5)=0.69\;\times \sum_{1=1}^{n}\int_{ai}^{bi}P(PM2.5)i\left(x\right)d(x)$$

$$EXP\;(PM10)=0.69\times \sum_{1=1}^{n}\int_{ai}^{bi}P(PM10)i\left(x\right)d(x)$$

where

i = is the number of days prior to the ophthalmic visit. The variation is from 1 to 10.

a, b is the temporal interval in hours, in which cyclists have used the bicycle in the “i” day.

Pi is the pollutants level detected by sensors with data transmissions each 5 s, calculated in terms of P2.5, P10.

0.69 is the adaptive coefficient.

*Statistics*—Clinical evaluations were performed on both eyes; however, for analyses related to particulate detection, only the right eye was considered, as it represented the source of the analyzed sample. For general clinical parameters unrelated to SEM analysis, the average of both eyes was used. For clinical parameters evaluated in both eyes, data from the right and left eye were averaged for each subject. This approach was adopted under the assumption of symmetrical exposure to environmental factors and to facilitate data interpretation in this pilot context. Data were analysed using MedCalc version 23.3.7. Normality of data distribution was assessed before statistical analysis. Continuous variables were expressed as median ± standard deviation (SD), along with minimum and maximum values. Spearman’s correlation coefficient (ρ) was calculated to assess associations between clinical parameters and individual exposure (EXP), ranging from −1.00 to +1.00. Correlation strength was interpreted as follows: very high (|R| = 0.85–1.00), high (0.60–0.85), moderate (0.30–0.60), low (0.20–0.30), and negligible (<0.20). A *p*-value < 0.05 was considered statistically significant. For SEM analysis, particle density was calculated as the average number of particles per field across 20 randomly selected, non-overlapping fields in the right-eye samples. Results were reported as mean ± SD. No inferential statistical testing was applied to SEM-EDS data due to the exploratory and descriptive nature of this imaging component.

## 3. Results

The demographic characteristics of the fifteen subjects enrolled are reported in Table 2.

Results recorded for subjects in terms of pollutant exposure, temperature and humidity are also reported in Table 2.

Results from clinical investigation are reported in Table 3.

**Figure 1:**
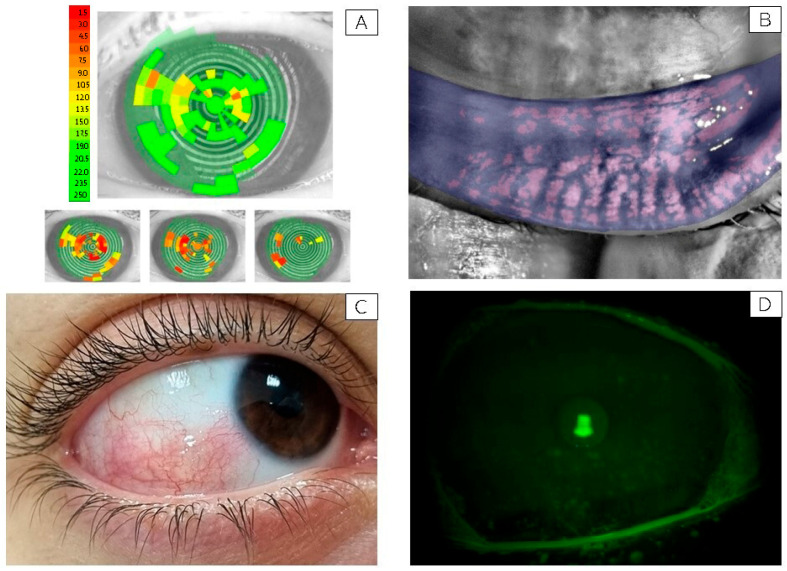
Representative ocular surface alterations observed in study participants. (**A**) Non-Invasive Break-Up Time (NiBUT) map of a representative subject with tear film instability (NiBUT = 5 s). The map displays the tear film breakup locations over time, color-coded from green (stable) to red (unstable), reflecting the seconds to first breakup after a complete blink. (**B**) Infrared meibography image showing approximately 50% loss of the meibomian glands in the lower eyelid. Glands are visualized using infrared light and false coloring, allowing evaluation of structural integrity and dropout areas. (**C**) Clinical image of the right eye from a representative subject with bulbar conjunctival hyperemia graded 3 on the Efron scale [25]. Picture taken with a mobile phone at the original magnification 5×. (**D**) Slit-lamp image under cobalt blue illumination showing fluorescein staining of the corneal surface in a subject graded NEI score 3 for corneal damage. Green fluorescein dots indicate areas of epithelial disruption [34]. Picture taken with a Topcon slit lamp equipped with a DC-4 camera, original magnification 16×.

A reduced tear film stability (NiBUT < 10 s) was observed in all participants, showing a non-significant inverse trend with PM2.5 and PM10 exposure levels. An example of a subject with markedly low NiBUT is shown in Figure 1A.

Meibomian gland loss showed a median depletion of approximately 40%, consistent with the reduced tear stability observed. A representative image of meibomian gland dropout is shown in Figure 1B.

Conjunctival hyperemia with an Efron score greater than 2 was observed in six out of fifteen participants. In this subgroup, a statistically significant, strong positive correlation was found between conjunctival hyperemia and exposure to both PM2.5 (R = 0.80, *p* = 0.0001) and PM10 (R = 0.611, *p* = 0.02). A representative image of bulbar hyperemia with an Efron score of 3 is presented in Figure 1C.

No significant correlation was found between conjunctival hyperemia and environmental temperature or humidity levels.

The Ocular Protection Index (OPI) and interblink interval (IBI) were within normal ranges in all participants.

Regarding subjective ocular discomfort, eight out of fifteen participants reported pathological OSDI scores, particularly driven by subscale A (ocular symptoms). The Numerical Rating Scale (NRS) for ocular pain showed a median value above 4 across all subjects. Moderate positive correlations were observed between overall OSDI scores and PM2.5 exposure (R = 0.35), as well as between OSDI subscale A and PM2.5 (R = 0.42) and PM10 (R = 0.31) exposures. A weak, non-significant inverse correlation was noted between NiBUT and PM2.5 levels (R = −0.259).

As for conjunctival damage, a significant positive correlation was found with both PM2.5 (R = 0.62, *p* = 0.02) and PM10 (R = 0.55, *p* = 0.04) exposures. Similarly, corneal damage showed strong correlations with PM2.5 (R = 0.719, *p* = 0.005) and PM10 (R = 0.65, *p* = 0.01). No significant associations were observed between conjunctival or corneal damage and ambient temperature or humidity levels. A representative image of a subject with corneal fluorescein staining graded NEI score 3 is shown in Figure 1D.

No other statistically significant correlations were identified among the remaining clinical parameters.

SEM-EDX analysis of dried tear samples from unwashed eyes revealed the presence of multiple microparticles embedded in the tear matrix. The particles displayed variable morphology and were clearly visible at 200× magnification. A total of 63 particles were detected and characterized through the three analyzed individuals. The number of particles recovered from each subject showed marked variability, with 14 particles identified in Subject 1, 19 particles in Subject 2, and 30 particles in Subject 3. Particles were irregularly distributed on the filter surface, showing heterogeneous morphology and size.

The majority of particles belonged to the PM2.5 fraction (1–2.5 µm), whereas PM10-sized particles (2.5–10 µm) were less represented, and PM1 particles (≤1 µm) were only occasionally observed, data are shown in Table 4. Morphometric descriptors calculated in ZEISS ZEN showed a broad variability in projected area and Feret diameters, with median (±SD) values across subjects of 3.2 ± 1.4 µm^2^ for area, 1.4 ± 0.6 µm for minimum Feret diameter, and 2.3 ± 0.8 µm for maximum Feret diameter. Circularity values ranged from 0.55 to 0.95 (median = 0.78), indicating that most particles were moderately rounded, although some irregular and elongated shapes were also observed.

Overall, these results indicate that the particulate matter recovered from the tear samples was predominantly composed of fine particles within the PM2.5 range, with variable morphology and a limited presence of coarse (PM10) or ultrafine (PM1) fractions.

**Figure 2:**
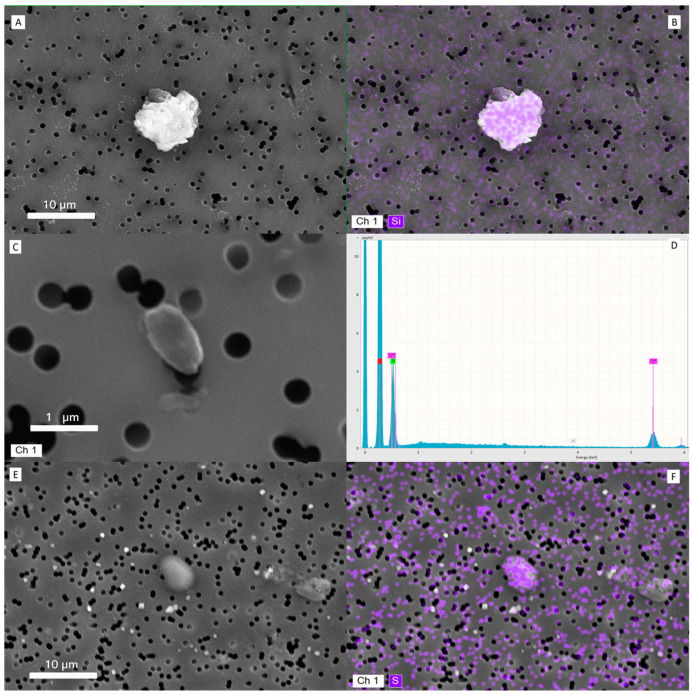
Representative images of particulate matters deposited onto filter and their characterization. (**A**) Scanning Electron Microscopy (SEM) image of a PM10 particle from a tear sample (subject #10). (**B**) Corresponding Energy-Dispersive X-ray Spectroscopy (EDS) elemental map showing a silica-based composition of the same particle. (**C**) SEM image of a PM1 particle identified in the tear matrix (subject #2). (**D**) EDS spectrum of the particle in (**C**), revealing predominant carbon content (red label), alongside chromium from the sputter-coating process. (**E**) SEM image of a PM5 particle with sulfur-rich composition, typical of atmospheric particulate matter. (**F**) EDS elemental map of the particle in (**E**), confirming sulfur as the major constituent.

In Figure 2, a collection of representative images. Particles were identified as carbon-rich agglomerates, metallic debris (primarily Fe, Si, and Al), and fibrous fragments, consistent with urban atmospheric particulate. In contrast, tears sampled from the washed control eyes showed no detectable particulate matter under the same conditions.

## 4. Discussion

Data from this small cohort suggest that specific parameters of the ocular surface may be altered in otherwise healthy individuals who regularly commute by bicycle in an urban environment and are chronically exposed to air pollutants, as estimated through personalized low-cost sensors. This approach allowed a fine-grained quantification of personal exposure, offering a significant advantage over conventional fixed-site monitoring systems.

Similar subject selection criteria—such as the exclusion of contact lens wearers, allergic individuals, and those with recent ocular surgery—have been adopted in previous studies on urban cyclists, to minimize potential confounding factors and ensure both ethical and methodological consistency in personal exposure designs [34,35].

Clinical evaluations were performed on both eyes, but for statistical purposes, we arbitrarily chose to use the average of the two eyes. The optimal statistical approach for analyzing data from paired organs such as the eyes remains a matter of ongoing debate and is yet to be definitively resolved [36]. While a commonly accepted, though suboptimal, method involves analyzing data from only one eye—either pre-specified or the more severely affected based on clinical judgement—we opted for averaging both eyes under the assumption that environmental exposure to airborne pollutants would be reasonably symmetrical.

In our cohort, the Ocular Protection Index (OPI) and interblink interval (IBI) were within normal ranges in all participants, as expected given the overall healthy status of the cohort, likely reflecting a preserved compensatory capacity against environmental stressor. Instead, tear film instability was evident in all subjects, as NiBUT values consistently fell below the 10-s threshold, suggesting a reduced capacity of the tear film to maintain surface integrity. This observation aligns with previous findings indicating that tear film is a sensitive indicator of early environmental stress on the ocular surface [12]. The reduction in NiBUT showed a weak, non-significant inverse trend with increasing exposure to PM2.5 and PM10, a finding that may reflect the multifactorial nature of tear film instability and warrants further investigation in larger populations.

Meibomian gland dysfunction, assessed by infrared meibography, was also observed, with a median gland loss of approximately 40%. This is a noteworthy finding considering the relatively young, healthy profile of the sample. Since the meibomian glands contribute to the lipid layer of the tear film, their depletion may partially explain the reduced NiBUT values observed, further supporting a possible environmental etiology.

Importantly, conjunctival hyperemia—an easily detectable clinical sign of ocular surface inflammation—was present in 6 out of 15 subjects. In these individuals, a statistically significant correlation was found between the degree of hyperemia and exposure levels to both PM2.5 and PM10, suggesting a dose-dependent relationship. This supports earlier research on the irritative potential of air pollution on mucosal surfaces, including the eye [13,18].

Corneal and conjunctival staining, as scored by the NEI grading scale, also correlated with PM exposure, particularly with PM2.5, highlighting the capacity of smaller particles to penetrate the tear film and trigger epithelial damage. Several biological pathways may explain the link between exposure to airborne particulate matter and ocular surface changes. For instance, fine particles can deposit on the tear film and conjunctiva, triggering local oxidative stress and generation of reactive oxygen species (ROS). These ROS can damage epithelial cells and lipid components of the tear film, leading to instability and increased evaporation. In addition, particulate exposure has been associated with up-regulation of pro-inflammatory cytokines (e.g., IL-6, IL-8) and activation of resident immune cells in the conjunctiva, resulting in hyperemia and epithelial staining. Furthermore, chronic exposure may compromise the densely innervated corneal surface, altering sensory feedback and contributing to the symptom–sign mismatch often observed in dry-eye-type conditions. These mechanisms are consistent with conclusions from recent research on the role of environmental conditions in ocular surface disease [12]. These clinical observations are biologically plausible, as PM exposure has been shown to induce oxidative stress, epithelial cell apoptosis, and increased production of inflammatory cytokines at the ocular surface, ultimately contributing to mucosal barrier disruption and discomfort [37,38]. These findings resonate with the emerging view that the ocular surface can serve as an accessible and non-invasive model to assess environmental toxicity in vivo [12,13].

Subjective symptoms, as assessed by the OSDI and pain NRS, were reported by more than half of the participants, primarily within the ‘ocular discomfort’ subdomain (subscale A), and showed moderate correlations with PM levels. This discrepancy between reported symptoms and clinical signs is characteristic of environmentally induced ocular surface disease and has been previously described in studies evaluating dry eye in polluted environments [12,13]. This symptom–sign mismatch is a well-documented feature of dry eye disease and other ocular surface disorders, where subjective discomfort does not always correlate with the severity of clinical findings. Possible explanations include individual variability in nociceptive sensitivity, subclinical inflammation, neurosensory alterations, and psychosocial influences [39].

Data from this preliminary study showed that pollutant concentrations during the observation period were consistent with the average levels recorded over the previous three years (2021–2023), according to the ARPA Emilia-Romagna database. Air temperature and humidity did not present any significant peaks during the study timeframe. These two physical parameters did not appear to influence ocular surface characteristics; however, since all clinical examinations were conducted under similar weather conditions, potential fluctuations related to temperature and humidity could not be assessed.

It is important to note that air quality in outdoor settings—such as bike commuting routes or urban streets—directly determines the quality of the surrounding micro-environment, including indoor, semi-outdoor and personal breathing zones. Indeed, outdoor pollutants can infiltrate buildings and enclosed spaces through ventilation, infiltration, or human movement, thereby contributing to the cumulative pollutant load experienced by individuals [40,41]. As our study focuses on real-life cycling exposure in an urban environment, this outdoor–personal linkage strengthens the public-health relevance of our findings.

Overall, the findings suggest that ocular surface examinations, combined with validated symptom questionnaires, may serve as a reliable proxy for evaluating the health impact of air pollution over short timeframes. In contrast to epidemiological studies—which typically capture outcomes such as increased morbidity or premature mortality over longer periods—the model proposed here could offer a more immediate tool for estimating the biological effects of environmental exposure.

SEM-EDX analysis of tear samples revealed the consistent presence of airborne-like microparticles embedded within the tear matrix, contrasting with the absence of detectable material in rinsed control eyes. This observation suggests that the tear film is directly exposed to and capable of transiently retaining environmental particulate matter. These findings support the hypothesis that the tear film may function as a short-term reservoir for airborne particles, although further investigation is required to characterize deposition dynamics, residence time, and the correlation with ambient exposure levels. Given that SEM-EDS was performed on only three tear samples, these findings are strictly exploratory and not generalizable.

Taken together, these data provide preliminary evidence that even moderate, chronic exposure to urban air pollutants can affect ocular surface homeostasis, leading to subclinical inflammation and symptom onset. Importantly, our findings show that both objective signs (e.g., hyperemia, gland loss, corneal staining) and subjective symptoms (OSDI) are sensitive to this environmental burden.

Apart from contributing to global datasets on personal air quality, BIKE EYE also introduces an innovative approach to evaluate the effects of the exposure to air pollutants, simultaneously proposing a new protocol for air pollution impact assessment. In fact, while most of the studies on air pollution impact focus on respiratory health, this project proposes the eye as a more accessible indicator of the inflammation caused by urban aerosol. Tears are emerging as an excellent biofluid for analysis, and an ideal source of biomarkers mirroring individual health status, since they are influenced both by changes in blood plasma and by the external environment. Although preliminary, this pilot project may lay the groundwork for future studies aiming to identify new markers of environmental sensitivity in lacrimal fluid, which is regarded as the “next routine body fluid test”, a less invasive alternative to blood test.

As reported, several papers published in the literature showed associations between air quality in specific polluted areas and the conjunctivitis prevalence in the population exposed. The novelty in this research refers to the direct observation and impact on a specific cohort of healthy subjects, measured in terms of amounts of pollutant exposure. Data suggest that the ocular surface—in particular, the conjunctiva, the only exposed mucosa in the body—may serve as a sentinel organ for environmental exposure, and tear fluid is increasingly recognized as a non-invasive biomatrix for monitoring pollutants [12,42,43].

However, the study has several limitations, particularly the low sample size since it is a preliminary pilot study, with just 15 subjects, although finely selected. Another limitation is related to the study design with only one-shot analysis; not being a longitudinal study, it was not possible to register variations in the ocular parameters depending upon different pollutants levels. Finally, there is a lack of certainty as to the univocal correlation between exposure and parameters, since we could not observe subjects before exposure to pollutants. The pilot study presents compelling evidence linking air pollution, specifically PM2.5 and PM10, to ocular surface damage and discomfort among subjects. The strong correlations raise important questions about environmental influences on health and drive us for future sampling of tears and cells to evaluate apoptotic and inflammatory biomarkers directly in an exposed and reactive mucosa—the conjunctiva—which could shed new light on the issue.

## Figures and Tables

**Table 1:** Sensor types.

Measurement	Units	Sensor
Air temperature	°C	Sensirion SHT-31
Relative Humidity	% RH	Sensirion SHT-31
Noise Level	dBA	Invensense ICS-434342
Ambient light	lx	Rohm BH1721FVC
Barometric pressure	kPa	NXP MPL3115A26
Equivalent Carbon Dioxide	ppm	AMS CCS811
Volatile Organic Compounds	ppb	AMS CCS811
Particulate Matter PM1/2.5/10	µ/m^3^	PlantPower PMS 5003

**Table 2:** Demographic and Environmental Exposure Characteristics of the Study Population.

Parameters	
Gender (males/females)	12 males, 3 females
Age (years)	46 (38–63) [44.5–52.8]
Pollutant exposure PM 2.5	17.7 (2.5–111.3) [6.9–65.5]
Pollutant exposure PM 10.0	33.6 (2.0–178.7) [6.7–80.2]
Temperature exposure	12.2 (9.8–15.7) [11.2–13.4]
Humidity (%)	66.0 (52.6–74.8) [55.5–69.6]

Data are expressed as median (min–max) [95% confidence interval]. Pollutant exposure is expressed in µg/m^3^·h. PM = particulate matter.

**Table 3:** Ocular surface clinical parameters.

Parameters	
Conjunctival hyperemia (Efron scale)	1.5 (0–3) [1,2]
Corneal damage (NEI)	2.0 (0–4) [0–2]
Conjunctival damage (NEI)	1.0 (0–3) [0–2]
Tear production (Schirmer test, mm/5 min)	15.0 (3.0–25.0) [11.0–20.0]
Tear stability (NiBUT)	6.0 (4.0–11.0) [4.7–10.0]
Interblink Interval (IBI)s	3.9 (2.2–5.8) [2.5–5.7]
OPI	1.9 (0.7–4.8) [0.9–4.2]
Meibomian gland loss (%)	36.6 (19.0–51.5) [29.9–43.3]

Data are expressed as median (min–max) [95% Confidence Interval]. NEI = National Eye Institute scale; NiBUT = Non-Invasive Break-Up Time; IBI = Inter-Blink Interval; OPI = Ocular Protection Index; mm = millimeters; min = minutes.

**Table 4:** Morphometric distribution of tear-borne particles detected by SEM.

Size Category	Mean Particle Count ± SD	Relative Proportion (%)
PM1 (≤1 µm)	2.7 ± 1.5	13%
PM2.5 (1–2.5 µm)	10.3 ± 4.2	52%
PM10 (2.5–10 µm)	7.0 ± 3.6	35%

Summary of the number and relative proportions of particles identified in tear samples from three individuals (n = 63 total particles; 14, 19, and 30 particles respectively per subject). Particles were classified according to their equivalent circle diameter (ECD) into three size categories: PM1 (≤1 µm), PM2.5 (1–2.5 µm), and PM10 (2.5–10 µm). Values are expressed as mean particle count ± standard deviation (SD) across the three subjects, together with their corresponding relative proportions (%).

## Data Availability

Data will be made available under reasonable request.
